# Mucin-induced metabolic reprogramming in *Pseudomonas aeruginosa* clinical isolates

**DOI:** 10.1128/msystems.00580-26

**Published:** 2026-07-02

**Authors:** Mohammad Mazharul Islam, Glynis L. Kolling, Joanna B. Goldberg, Katharina Ribbeck, Jason A. Papin

**Affiliations:** 1 Department of Biomedical Engineering, University of Virginia 2358, Charlottesville, Virginia, USA; 2 Department of Pediatrics, Emory University 1371, Atlanta, Georgia, USA; 3 Department of Biological Engineering, Massachusetts Institute of Technology 2167, Cambridge, Massachusetts, USA; Universita degli Studi di Verona, Verona, Veronai, Italy

**Keywords:** *Pseudomonas aeruginosa*, transcriptomics, mucin, metabolic reprogramming

## Abstract

Mucins modulate microbial metabolism, which is crucial in many human infections. It is unknown whether the metabolic alterations induced by mucin are universal or unique across varied clinical isolates of bacterial pathogens. We experimentally profiled a representative set of seven clinical *Pseudomonas aeruginosa* isolates in a synthetic cystic fibrosis growth medium in the presence or absence of physiological mucin, using high-throughput transcriptomic analyses. We observed mucin-induced transcriptional changes across many central and peripheral metabolic pathways in all the clinical isolates. We utilized contextualized metabolic network models of *P. aeruginosa* clinical isolates to gain a deeper understanding of the relationship between mucin-driven metabolic modulations and the resulting shifts in metabolic dependencies. Our network model-driven analyses and subsequent growth experiments reveal differential effects of mucin on isolate metabolism and growth inhibition of key genes. Characterizing this rich set of *P. aeruginosa* isolates allows for a deeper understanding of the diversity of the pathogen and how mucins modulate isolate-specific pathways that may significantly impact treatment strategies.

**IMPORTANCE:**

Mucin, the principal component of mucus, is a key regulator of host-microbe interactions and substantially influences *P. aeruginosa*, a major antibiotic-resistant pathogen. While mucins are known to influence microbial physiology, most bacterial physiology studies rely on reference strains or mucus-free systems, failing to capture the complexity of host-associated environments and clinical isolate diversity. To address these shortcomings, we used purified mucin from porcine lung and a physiologically relevant medium to examine seven clinical isolates of *P. aeruginosa*. Transcriptomic profiling and genome-scale modeling revealed both universal and isolate-specific mucin-driven metabolic shifts, identifying pathways critical to adaptability and candidate drug targets. These findings highlight mucin as an active influencer of *P. aeruginosa* metabolism and underscore its potential implications for developing more effective, context-specific infection treatments.

## INTRODUCTION

The relationship between mucin, the primary component of mucus, and *Pseudomonas aeruginosa* is critical for understanding many disease outcomes. For example, *P. aeruginosa* is highly prevalent in the lungs of cystic fibrosis patients, in which dysregulated mucin production and decreased clearance are observed (1). While some studies have explored these relationships (2), it is important to deepen our understanding of the mechanistic interactions between mucins and *P. aeruginosa*. Furthermore, *P. aeruginosa* exhibits distinct responses to specific mucin molecular structures compared to other analogous polymers (3–5), supporting the observation that the specific glycan structures on mucin have signaling functions that directly influence microbial metabolism and virulence factor activity.

*Pseudomonas*-mucin interactions during infection in the human body have been explored in several past studies (3, 5–9). These interactions lead to metabolic reprogramming, which can play an important role in health, disease progression, recovery, and treatment. However, to date, our understanding is limited to a cataloging of the components in this complex milieu with little understanding of the mechanisms underlying the metabolic phenotypes that emerge from the interactions of microbes and mucins. In addition, many such studies were focused on the interaction of mucin components with standard laboratory strains of *P. aeruginosa*, namely PA01 and PA14. In a recent study, we demonstrated that clinically isolated strains possess distinct metabolic capacities, which manifest as diverse functional capabilities and morphologies (10). We hypothesize that the functional diversity within a representative set of clinical isolates will influence the response to the presence of mucin. Whether the influence of mucin is observed universally across all the clinical isolates or only uniquely by a subset of the isolates has profound implications in designing an effective treatment strategy in clinical settings.

To understand the extent and diversity of mucin-driven metabolic shifts in clinical *P. aeruginosa* isolates, we performed a detailed transcriptomic study and genome-scale metabolic network model-based analyses of seven clinical *P. aeruginosa* isolates. We used isolates from our previous deep profiling study of a representative group of clinical *P. aeruginosa* isolates (11). We cultured the isolates in a synthetic cystic fibrosis medium (SCFM2) in the presence and absence of mucin. We identified differentially expressed genes and enriched pathways in the presence of mucin. We also performed *de novo* transcriptomics to identify changes in the unique metabolic genes in the clinical isolates that do not map to the reference genome of *P. aeruginosa* strain PA14. Mucin-modulated functional changes appear distinct between the isolates. We also constructed contextualized genome-scale metabolic network models of the clinical isolates based on the transcriptomic data to predict genes that play a crucial role in the modulation of microbial metabolism in the presence of mucin.

## RESULTS AND DISCUSSION

### Clinical *P. aeruginosa* isolates were selected to capture phenotypic and functional diversity

Between February 2019 and February 2020, a total of 971 clinical *P. aeruginosa* isolates from 590 patients at the University of Virginia (UVA) Health System were collected, as reported previously (11). For each of the 971 clinical *P. aeruginosa* isolates, patient demographic profiles (age and sex), comorbidities (cystic fibrosis or diabetes), and isolate phenotypic traits (mucoid phenotype, metallic sheen, pigment production, and hemolytic activity) were collected and tabulated. To understand their genotypic, phenotypic, and metabolic variance and assess the shared and unique traits that they can manifest, we extensively studied a representative sample population of 25 of these *P. aeruginosa* isolates in previous work (10). We explored the genomic, sequence type, and phenotypic diversity within the representative set of isolates. While the whole genome sequencing and annotation provide us with a rich picture of the functional capabilities of the clinical isolates, here, we selected seven isolates representing their phylogenetic and phenotypic diversity for further transcriptomic analyses with the goal of characterizing their metabolic adaptations. A table summarizing each isolate, its origin, patient metadata, and relevant clinical features is included in Information S1. Functional transcriptomics of the core and unique metabolism of the different isolates will help enable the evaluation of the strain-specific variations in virulence mechanisms and adaptability.

### Differential gene expression induced by mucin is observed universally across all isolates

Mucins are the primary macromolecules in mucosal layers and are known to modulate microbial phenotypes (3, 8, 12–14). To investigate how the metabolism of *P. aeruginosa* shifts when in the presence of mucins, we cultured seven clinical *P. aeruginosa* isolates in a customized SCFM2 (see Materials and Methods) in the presence and absence of mucin. Total RNA was isolated and sequenced (see Materials and Methods for details). After processing the raw transcriptomic data, gene counts in SCFM2 and SCFM2 + mucin were calculated and used for differential gene expression analysis to identify mucin-responsive genes in all seven clinical *P. aeruginosa* isolates as well as the reference strain PA14. Genes with a log_2_ (fold change) value of 1.5 or higher were considered overexpressed, and genes with a log_2_ (fold change) value of −1.5 or lower were considered underexpressed while satisfying an adjusted *P*-value of <0.05. See Information S2 for a complete list of all the differentially expressed genes in the presence of mucin in each isolate.

**Fig 1:**
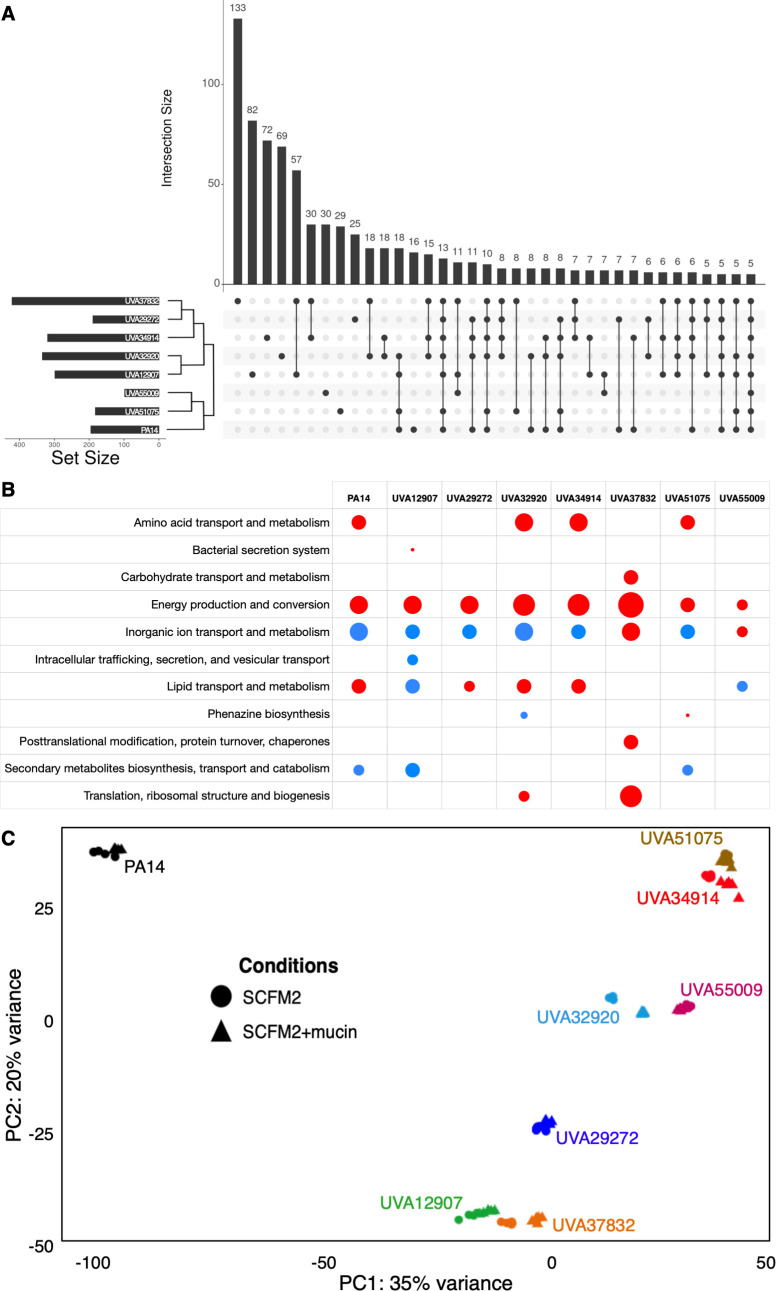
Differential gene expression in *P. aeruginosa* clinical isolates in the presence of mucin. (**A**) Distribution of differentially expressed genes across the isolates is shown in UpSet plots. Each of the vertical bars represents the number of significantly differentially expressed genes, and the distribution across different isolates is represented by the solid circles across the isolate names at the bottom. Isolates are vertically arranged based on their phenotypic clustering tree, which is shown beside the bars for the set size for each isolate. (**B**) Significantly enriched pathway subsystems in the presence of mucin across clinical *P. aeruginosa* isolates and the reference PA14 strain. Circle sizes represent the number of differentially expressed genes in each pathway subsystem in SCFM2 + mucin vs. control (SCFM2). Red indicates that the genes are overexpressed, and blue indicates that the genes are underexpressed. (**C**) Principal component analysis (PCA) plots of gene expression profiles of *P. aeruginosa* isolates, including PA14. Mucin-induced metabolic changes are less prominent compared to inter-isolate differences.

The most dominant pathways differentially expressed across all or most of the clinical isolates are energy production and conversion, and amino acid transport and metabolism (Fig. 1). Consistently overexpressed pathways across nearly all the clinical isolates include amino acid transport and metabolism, energy production and conversion, and lipid transport. Genes involved in glyoxylate/dicarboxylate metabolism; glycine, serine, threonine metabolism; and nitrogen metabolism were also consistently upregulated in mucin across many isolates. Inorganic ion transport and metabolism, as well as secondary metabolite biosynthesis and catabolism, showed significant downregulation in the presence of mucin. Only five genes were underexpressed in all of the clinical *P. aeruginosa* isolates, as well as the PA14 strain.

Unique profiles in certain pathways and localization categories provide potential targets for therapeutic interventions. High variation in the expression of energy production genes suggests that isolates exhibit differential metabolic states, possibly linked to strain-specific environmental adaptation or host interaction. The dominance of cytoplasmic and membrane-associated genes highlights their central role in pathogenicity and resistance. In addition, another major group of genes differentially expressed as a function of mucin presence is annotated with either unknown or hypothetical functions, which underscores the need for further functional characterization.

Commonly overexpressed genes in the presence of mucin included those involved in biofilm formation, motility, and secretion systems. Notably, the *fleQ* gene, a transcriptional regulator of flagellar synthesis, was overexpressed in several isolates. Mutations in *fleQ* result in loss of motility and reduced mucin adhesion, impairing the ability of *P. aeruginosa* to colonize host tissues (15, 16). This interaction underscores the bacterium’s adaptability to mucosal environments. While we do not observe statistically significant overexpression or underexpression of genes related to flagellar motility, as investigated in our earlier works (3, 8), a response in *fleQ* in the presence of mucin indicates a virulence-attenuating change that can be parallel to changes in motility of aggregation. Genes associated with acute virulence factors, such as those coding for type III secretion system components, were underexpressed in certain isolates, indicating a potential shift toward a chronic infection phenotype.

Nitrate and nitrite reductases and nitrate-inducible formate dehydrogenase subunits were overexpressed in the presence of mucin. The denitrification pathway enables *P. aeruginosa* to perform anaerobic respiration. This capability is particularly important in oxygen-limited environments, such as the thick mucus present in the lungs of cystic fibrosis patients. Nitrate-inducible formate dehydrogenase (*fdnH*) has been previously shown to be significantly upregulated when interacting with primary normal human airway epithelial cells (17) and during growth in CF sputum (18). The ability to respire anaerobically via denitrification allows *P. aeruginosa* to survive and persist in hypoxic conditions, contributing to chronic infections (19).

Sarcosine oxidase subunits, including *soxD* and *soxB*, were overexpressed in four isolates. These genes are implicated in sulfur metabolism and are essential for energy production and virulence, and could serve as potential therapeutic targets. In addition, several lipase enzymes, including *lipC*, were upregulated in most of the isolates. These enzymes potentially aid in nutrient acquisition and tissue invasion during infection by hydrolyzing lipids. Lipases have been implicated in the pathogenicity of various bacteria by breaking down host cell membranes and modulating immune responses (20, 21). Although direct evidence linking *lipC* to *P. aeruginosa* infections is limited, the general role of lipases in bacterial virulence supports its potential involvement.

Many genes involved in inorganic ion transport and metabolism were underexpressed in the presence of mucin across all isolates except one, UVA37832. Among them were the molybdate-binding periplasmic protein precursor (ModA) and molybdenum transport protein (ModB), which are crucial for the uptake of molybdate ions. Molybdate is a vital cofactor for various enzymes involved in metabolic processes. While specific studies directly linking *modA* to *P. aeruginosa* pathogenicity are limited, the role of molybdate in bacterial metabolism suggests that *modA* could influence the bacterium’s adaptability and virulence (22). *P. aeruginosa* T6SS-mediated molybdate transport contributes to bacterial competition during anaerobic growth (23). Superoxide dismutase (encoded by *sodM*) protects the cell from oxidative stress by neutralizing reactive oxygen species. Oxidative stress resistance is crucial for bacterial survival within the host, as immune cells generate reactive oxygen species to kill pathogens. While specific studies on *sodM* in *P. aeruginosa* are scarce, superoxide dismutases are generally recognized as important virulence factors in many pathogens (24).

Overall, mucin exposure induces transcriptional changes in *P. aeruginosa* clinical isolates discussed above, marked by upregulation of energy production, amino acid metabolism, and biofilm-associated genes, supporting adaptation to mucosal environments and downregulation of inorganic ion transport and acute virulence. Together, these findings not only deepen our understanding of *P. aeruginosa* pathophysiology but also point to promising targets for therapeutic intervention, especially among genes with uncharacterized functions or variable expression across clinical isolates.

### Clinical isolates are differently affected by mucin compared to the reference PA14 strain

While mucin-induced metabolic shifts are observed in every isolate and span both central and peripheral metabolism, it was observed that each isolate was differentially affected by mucin. The number of significantly differentially affected genes (*p*_adj_ < 0.05) varied among the isolates. Figure 1A shows the number and overlap of differentially expressed genes across the isolates and the reference PA14 strain. Figure S1 and Fig. S2 show the UpSet plots for overexpressed and underexpressed genes, respectively. The greatest influence of mucin was observed in isolate UVA37832, which manifested 268 overexpressed genes and 153 underexpressed genes in the presence of mucin. On the other hand, the isolate differentially expressing the least number of mucin-induced genes was UVA55009, which had 60 overexpressed genes and 38 underexpressed genes in the presence of mucin. These mucin-induced metabolic shifts encompass both central and peripheral pathways. Figure 1B shows a pathway enrichment profile for each of the isolates.

Figure 1C shows a principal component analysis (PCA) plot of the isolates based on their gene expression profile, where the isolates show a noticeable spatial separation from the reference strain PA14, denoting that clinical isolates show distinct metabolic behavior in both SCFM2 and SCFM2 + mucin media compared to the laboratory strain. While the median Euclidean distance between the clusters of replicates in one isolate and another is 55.65, the reference strain PA14 cluster sits at a Euclidean distance of 117.55 from the clinical isolate cluster. This result demonstrates the importance of studying the metabolic reprogramming of pathogens in physiological settings using clinical strains and biologically derived media components like mucin.

### Mucin-induced metabolic changes are less prominent compared to inter-isolate differences

The PCA plot also shows that mucin-induced metabolic changes, denoted by the circular (SCFM2, no mucin) and triangular (SCFM2 + mucin) shapes, are less prominent compared to inter-isolate differences, denoted by the different colors (Fig. 1C). Please note our omission of mucin from the medium composition, as reported in previous studies (25), to study the effects of the presence and absence of mucin in the growth medium (SCFM2). The median Euclidean distance between the cluster of replicates in one isolate and another is 55.65, while within an isolate, the average distance between the samples grown in SCFM2 with and without mucin is only 5.88. This result demonstrates the complexity in the development of a decisive strategy to treat *P. aeruginosa* infections universally, since, although the physiological changes resulting from the effect of mucin are prominent; the metabolic diversity among clonal subtypes is of greater concern when devising an appropriate treatment regimen against *P. aeruginosa*.

### Mucin has a significant impact on genes unique to the clinical isolates (unmapped to PA14)

Each of the clinical isolates has unique functional capabilities encoded in its genome. While isolates share the majority of their genome with the reference *P. aeruginosa* strain PA14, 1.7%−4% of the genome sequences of the clinical isolates did not map to the reference PA14 strain. To extract, annotate, and quantify the expression of these unmapped genomic functions, we performed *de novo* transcriptome analysis on the unmapped sequences. *De novo* transcriptomic analysis is a vital approach for exploring the RNA landscape of organisms, especially those with uncharacterized or partially characterized genomes (26). This method bypasses the need for a reference strain, enabling the assembly of transcript sequences directly from raw RNA sequencing data. It is particularly valuable in studying complex pathogens like *P. aeruginosa*, where strain-specific variations may contribute to pathogenicity. By identifying differentially expressed genes, including those previously unmapped, *de novo* transcriptomics may provide insights into the molecular mechanisms underlying virulence, metabolic adaptation, and resistance. This study leverages *de novo* transcriptomic analysis to explore the gene expression profiles of *P. aeruginosa* strains under mucin-influenced conditions, highlighting unique and conserved pathways critical to infection and potential therapeutic targets. A visual schematic of our *de novo* transcriptomics pipeline is presented in Fig. S3.

The list of differentially expressed genes among the unmapped genes is presented in Information S3. While approximately 30%−55% of the differentially expressed coding sequences could not be mapped to a specific metabolic function, the rest of the unmapped (to the reference PA14 genome) genes, comprising many central and peripheral metabolic pathways, manifested mucin-induced differential expression.

**Fig 2:**
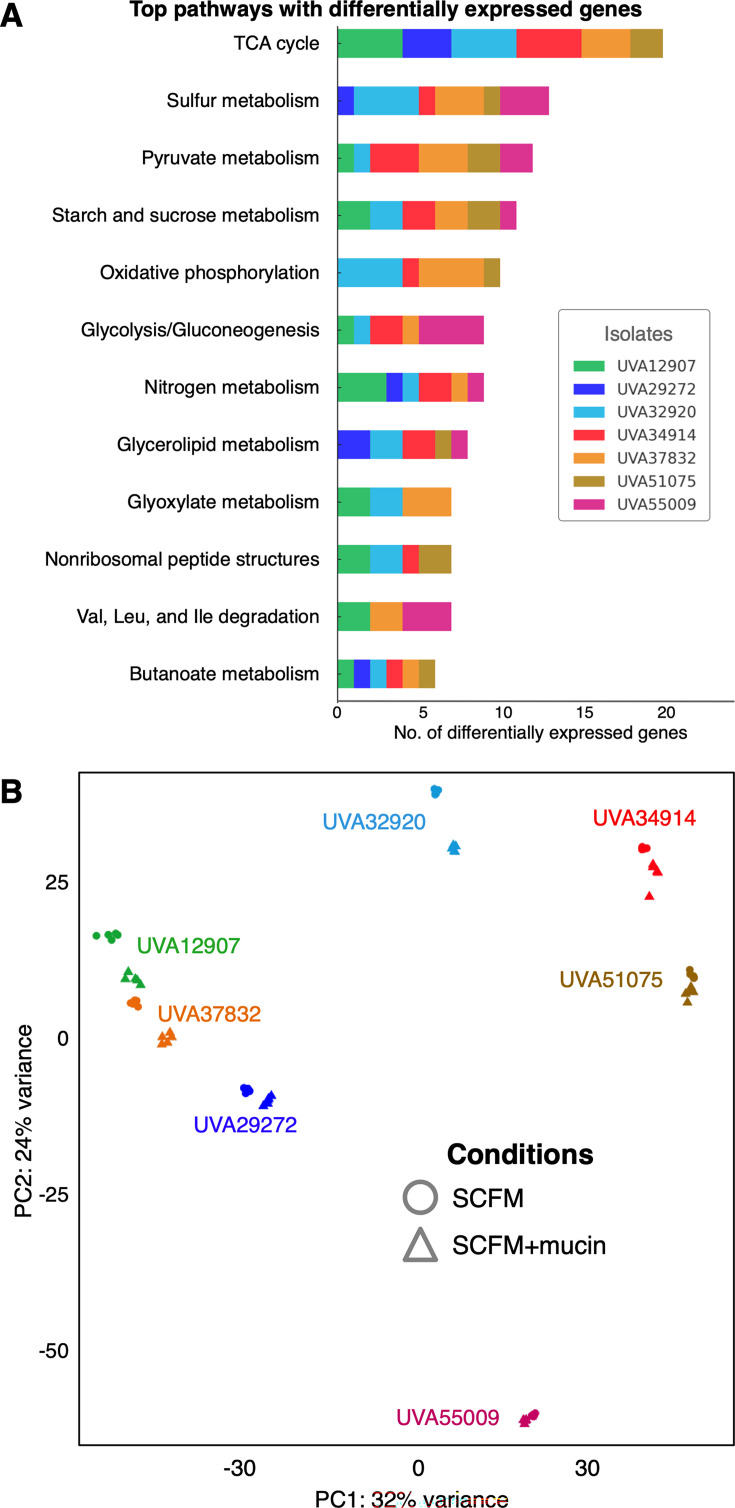
The diversity in mucin-influenced gene expression across *P. aeruginosa* clinical isolates. (**A**) Top pathways for unique differentially expressed genes unmapped to the reference PA14 strain across all isolates. Each isolate is represented by a different color. (**B**) PCA plot of gene expression profiles of *P. aeruginosa* isolates, including both the set of genes mapped to PA14 and the unmapped genes. Mucin-induced metabolic changes are less prominent compared to inter-isolate differences. Also, the effect of mucin is observed to be of varying degrees across clinical isolates.

Several key metabolic pathways, as shown in Fig. 2, notably the citrate cycle (TCA cycle), pyruvate metabolism, and glycolysis, have been prominently enriched in differentially expressed genes in all the isolates in the presence of mucin. The prominence of the TCA cycle among the unique DEGs suggests a significant shift in the isolates’ energy production mechanisms. Alterations in TCA cycle activity can influence the overall metabolic state, affecting not only energy production but also the synthesis of precursors for various biosynthetic pathways. Such changes may reflect adaptations to environmental stresses or shifts in nutrient availability, necessitating a reprogramming of metabolic fluxes to maintain cellular homeostasis. The identification of unique differentially expressed genes associated with pyruvate metabolism and glycolysis indicates potential modifications in how these isolates process glucose and manage energy production. Such alterations could be responses to hypoxic conditions, changes in nutrient availability, or other environmental factors that challenge the cells’ metabolic flexibility. By modulating glycolytic flux and pyruvate utilization, the cells can adapt to ensure survival and maintain function under varying conditions.

The enrichment of differentially expressed genes involved in these central metabolic pathways underscores the dynamic nature of cellular metabolism in response to external stimuli. Further investigations are warranted to elucidate the specific regulatory mechanisms governing these changes. Understanding the interplay between these metabolic pathways and gene expression not only sheds light on the fundamental metabolic adaptations and potential physiological responses of *P. aeruginosa* clinical isolates but also has potential implications for developing interventions in contexts where metabolic dysregulation is a hallmark.

### Contextualized metabolic network models predict metabolic adaptations and changing dependencies of *P. aeruginosa* clinical isolates in response to mucin

The integration of transcriptomic data with metabolic network reconstructions provides a greatly improved understanding of shifts in pathway utilization (27). Given the complex interplay of environmental factors, gene expression states, and metabolic phenotypes, computational models can be developed that predict how changes in gene expression lead to changes in metabolic functionality. Analysis of the network structure and flux distribution through different pathways in these models, contextualized by transcriptomic data, offers insight into the metabolic shifts in clinical *P. aeruginosa* isolates in the presence of mucins.

The flux sampling simulations of the transcriptomic data-integrated models of the isolates show significant differences in metabolic behavior, as seen in the non-metric multidimensional scaling plot in Fig. S4A. Each of the clinical isolates is distinguishable from the other, with a median Euclidean distance of 0.11 from the nearest neighbor. In addition, most of the isolate flux sampling results in both SCFM2 medium and SCFM2 + mucin cluster together. Similar to the transcriptomic profiles, the effect of mucin is less prominent than inter-isolate differences.

We simulated the contextualized network models to probe the effect of gene knockouts in the clinical *P. aeruginosa* isolates in SCFM2 media with and without mucin (see Materials and Methods for details). The threshold for essentiality was set at 10% of the wild-type growth (a 90% reduction in growth). A total of 173 reactions, encoded by 90 genes, are predicted to be essential for growth in the reference PA14 strain in both media. The complete list of these reactions is present in Information S4. Out of the 173 reactions that are essential in PA14, 164 are also essential in all the clinical isolates. The remaining nine reactions, which are catalyzed by eight genes (*rpiA, rpe, ndk, ispG, cmk, gltX, galU*, and *hemA*), are only essential in PA14. Some of these genes, identified as essential in PA14, are predicted to play key roles in metabolism, survival, and pathogenic behavior. For example, *rpiA* and *rpe* encode ribose-5-phosphate isomerase A and ribulose-5-phosphate epimerase, respectively, catalyzing steps in the non-oxidative branch of the pentose phosphate pathway. This pathway supplies ribose for nucleotide synthesis and NADPH for antioxidant defense. The essentiality of these genes in *P. aeruginosa* due to the incomplete EMP pathway is well known (28), which indicates a possibility of the clinical isolates utilizing uncharacterized redundant routes to complete sugar metabolism and therefore being robust against genetic perturbations.

Knocking out the reaction rxn03917, encoded by the gene *gmhA* (PA14_57500), predicted reduced growth in all of the clinical isolates, but not in PA14. The *gmhA* gene in *P. aeruginosa* encodes the enzyme D-glycero-D-manno-heptose 7-phosphate isomerase. This is a pivotal catalyst in the biosynthesis of the inner core of lipopolysaccharide (LPS). LPS is a fundamental component of the outer membrane in gram-negative bacteria, contributing to structural integrity and defense against environmental stresses, antibiotic susceptibility, and interactions with host immune systems (29). While the reference strain PA14 model contains alternate routes for the conversion of sedoheptulose 7-phosphate to D-glycero-D-manno-heptose 7-phosphate, the clinical isolates do not; thereby rendering the knockout of *gmhA* lethal in the clinical isolate model simulations. Given its central role in LPS formation and the outer membrane’s integrity, *gmhA* can be considered a potential target for novel antibacterial strategies aimed at compromising the protective barrier of *P. aeruginosa*, rendering it more susceptible to host defenses and antimicrobial agents.

Across all the clinical isolates, the addition of mucin in the SCFM2 growth media did not result in any change in essential gene predictions. However, mutations or knockouts in a few genes in *P. aeruginosa* clinical isolates predicted varying degrees of growth reduction in the presence of mucin, which are not completely lethal. It is to be noted that while genome-scale metabolic networks perform well in predicting gene essentiality (30), minor growth inhibitions upon gene knockouts are often low-confidence predictions. These genes, whose knockout predicted reduced growth in the presence of mucin, are shared across varying numbers of clinical *P. aeruginosa* isolates. One notable example is the knockout of rxn00260 (gene *aruH,* PA14_65770) that is predicted to inhibit growth between 50% and 85% of the wild-type growth in PA14 and in the isolates UVA29272, UVA32920, UVA51075, and UVA55009 in the presence of mucin. The gene *aruH* in *P. aeruginosa* encodes an arginine: pyruvate transaminase. This is the first catalytic enzyme of the arginine transaminase pathway for L-arginine utilization in *P. aeruginosa*. This pathway is only used when the major route of arginine catabolism, that is, the arginine succinyl transferase pathway, is blocked (31). Indeed, some of the downstream enzymes in these isolates, including succinylornithine transaminase and succinylglutamate desuccinylase, are inactive in the presence of mucin. The *aruH* gene has been historically annotated as aspartate transaminase (*aspTA*) due to high sequence similarities with other bacterial aspartate transaminase genes (31). However, biochemical characterization studies have concluded that this gene is indeed not an aspartate transaminase but rather an arginine: pyruvate transaminase (*aruH*), albeit having lessened substrate specificity to some other amino acids (31).

We attempted to assess the effects of loss of a gene function on the fitness of the clinical strains in the presence of mucin. Given there are no mutants available for the clinical isolates in this study, we decided to study the effects of mutations in these genes from the *P. aeruginosa* PA14 transposon mutant library (32). Out of the many essential genes and genes whose knockout resulted in reduced growth in our model simulations, we identified four mutants, namely, PA14_38350 (*galU*), PA14_65770 (*aruH*), PA14_72870, and PA14_11030, that were available in the mutant library. The other genes discussed above were not available in the mutant library, possibly because the other mutants were not viable or otherwise lethal during the transposon mutagenesis. The latter two of the four genes tested encode for an aminotransferase and had two separate transposon mutants in the library. The results are included in Fig. S4B.

We did not observe significant growth inhibition in SCFM media containing Muc5AC compared to SCFM media without mucin for any of the four genes tested. In addition, the wild-type PA14 exhibited the same growth rate in the presence and absence of mucin in the media. This result indicates that while the influence of mucin is observed in genome-wide redistribution of reaction fluxes, the overall fitness and metabolic dependencies do not change in a significant manner.

### Conclusions

Mucins are the key secreted component that make up mucus, which creates an interface between the pathogen and the host during infection. While the structural, regulatory, and diagnostic role of mucins has been reported in many studies, the effect of mucins on pathogen metabolic behavior has not been well explored. Moreover, as microbial pathogenesis is often studied in mucus-free environments that lack microbial interactions in physiological mucus gels, these results create a gap in our understanding of host-associated factors in bacterial pathogen colonization. We made deliberate use of purified mucin in our studies to begin to understand the roles of these key glycoproteins. Porcine mucin isolated from pig stomachs in our previous work (33) showed that it predominantly contains MUC5AC and small amounts of other mucins like MUC2, MUC5B, and MUC6. Previous studies also suggest that the MUC5AC from porcine mucin is homologous to the human glycoprotein and has viscoelastic properties similar to native mucus (34, 35). Bovine mucus was used as the source because of the obvious challenge to obtain sufficient levels of mucin from humans. Moreover, studies focused on reference strains of pathogens often fall short in capturing the vast functional landscape of clinically isolated *P. aeruginosa*, as is evident from our previous studies (10). To overcome this shortcoming, here, we performed deep transcriptomic profiling and subsequent model-based analyses of a representative group of *P. aeruginosa* clinical isolates in a physiologically relevant medium (22) in the presence (or absence) of mucin. The differential expression analysis identified both shared and isolate-specific effects of mucin in *P. aeruginosa* clinical isolates. Consistently overexpressed genes, such as *soxD*, represent promising targets for broad-spectrum therapies, while other isolate-specific genes offer insights into specific infection dynamics. Variations in energy metabolism suggest pathways critical to its adaptability. Our computational model-driven prediction of the key metabolic genes that modulate mucin-driven metabolic phenotypes is an efficient approach for identifying the mechanistic dependencies of genes. Growth assessment of PA14 mutants from the *P. aeruginosa* transposon mutant library revealed that there is much to be explored in the computational modeling and experimental validation study of mucin-induced changes in clinical isolates to achieve a clearer understanding of their metabolism. Future work could focus on the development of mutants for specific clinical isolates, reevaluation of model predictions, and exploring their clinical relevance.

## MATERIALS AND METHODS

### Isolation of porcine lung mucins

Isolation of porcine lung mucins was performed utilizing previously published methods from our group (36). Briefly, mucus was scraped from tissue and solubilized in NaCl buffer with protease inhibitors and sodium azide. Insoluble material was pelleted by ultracentrifugation, and mucins were purified using size-exclusion chromatography via cross-linked agarose gel filtration base matrix. Remaining contaminants were removed from preparations via CsCl gradient centrifugation. Mucin fractions were then desalted and lyophilized for long-term stability.

### Bacterial isolates and growth

Clinical isolates previously described in Dunphy et al. (11) were chosen as outlined in our previous study (10). All isolates were grown in SCFM2 (25) with the following modifications: DNA was omitted from the recipe due to interference with downstream RNA isolation, and porcine mucin was replaced with 0.5% wt/vol purified porcine Muc5AC (3). Isolates were cultured in modified SCFM2 overnight with shaking (190 rpm, 37°C) as a starter culture. Cultures were pelleted (5,000 × *g*, 3 min) and washed in fresh SCFM2; OD_600_ was measured and adjusted to a final density of 1.0. Cultures were added to fresh SCFM2 ±0.5% wt/vol Muc5AC in a 12-well culture plate to a final density of 0.1 (*n* = 5 replicates per condition). Plates were then incubated for 5 h at 37°C without shaking. For transposon mutant experiments, select mutants were grown overnight in LB and stocked prior to overnight growth in SCFM1 with the addition of Muc5AC as described above (Synthbiome, Atlanta, GA) at 37°C. The following day, OD_600_ was measured, and cultures were adjusted to an OD of 0.1 in fresh SCFM with or without 0.5% Muc5AC in a 48-well plate and incubated at 37°C for 5 h without shaking. Cell numbers were enumerated using plate counts in triplicate.

### RNA extraction and sequencing

An equal volume of ice-cold acetone:ethanol (1:1) was added to each culture to stabilize the RNA, and the plates were gently agitated. The full volume was then centrifuged to pellet bacteria (3,300 *× g*, 2 min), and the cells were pretreated with lysozyme (1 mg/mL, 2−3 min, RT) with gentle agitation. Total RNA was then isolated according to the manufacturer’s directions (Monarch Total RNA Miniprep Kit, NEB). In cases where an isolate was mucoid, the polysaccharide lyase Smlt1473 was added to lysozyme (0.15 mg total protein) prior to the addition of RNA lysis buffer (37). RNA was quantified using the Qubit RNA BR Assay Kit on a DeNovix fluorometer, and RNA quality was assessed using the Agilent TapeStation (UVA Genome Analysis and Technology Core, RRID:SCR_018883). RNA-seq was performed by SeqCenter LLC. (Pittsburgh, PA) using a package of 12 M paired-end reads/sample.

Samples were DNAse treated with Invitrogen DNAse (RNAse-free). Library preparation was performed using Illumina’s Stranded Total RNA Prep Ligation with Ribo-Zero Plus Kit and 10 bp IDT for Illumina indices. Sequencing was done on a NextSeq2000, generating 2 × 51 bp reads. Demultiplexing, quality control, and adapter trimming were performed with bcl-convert (v3.9.3).

### Differential expression and pathway enrichment analysis

Differential gene expression analysis was used to identify the mucin-induced changes from the transcriptomic data. The genes with a standard deviation in gene expression levels (read counts) less than 2% across all data points were discarded from the analysis. The DESeq2 algorithm (version 1.44.0) in the R (version 4.4.1) software package “Bioconductor” was used for differential gene expression analysis (38, 39). DESeq2 employs a negative binomial distribution and a shrinkage estimator for the distribution’s variance methods to test for differential expression. The raw read counts were used to calculate the fold change and the log_2_ (fold change) of the genes. Genes with a log_2_ (fold change) value of 1.5 or higher were considered overexpressed, and genes with a log_2_ (fold change) value of −1.5 or lower were considered underexpressed while satisfying an adjusted *P*-value of <0.05. Pathway enrichment analysis was performed to identify significantly enriched pathways in the presence of mucin. Pathway enrichment analysis was performed to identify significantly represented pathways among differentially expressed genes. Pathways with a false discovery rate (FDR) ≤ 0.05 were considered enriched.

### *De novo* transcriptome assembly and mapping

We used STAR aligner (version 2.7.10b, from https://github.com/alexdobin/STAR/releases, accessed May 2023) to align the isolate transcriptome sequences to that of the reference PA14 strain (40). From the STAR aligner sequence alignment, sequences not mapped to the reference PA14 were extracted as unmapped raw sequence files. Unmapped reads were output into the SAM Aligned.* file(s) with the “–outSAMunmapped” and .mate files formatted similar to .fastq files with the “–outReadsUunmapped” argument. Trinity (41) version 2.13.2 was used for *de novo* transcriptome analysis, including clustering of the unmapped reads and *in silico* read normalization. The built-in tool Jellyfish was used to build a k-mer (*K* = 25) catalog from reads, followed by linear contig assembly by Inchworm and contig clustering by Chrysalis. After harvesting all the assembled transcripts into a single multi-FASTA file, Salmon (version 1.10.2) was used to quantify the reads using Bowtie version 2.2.9. A blastx search was performed to annotate the coding sequences to the Uniprot (42) protein database (accessed December 2023).

### Genome-scale metabolic network modeling, contextualization, and analyses

To quantify the functional impact of these differences in metabolic gene content, we generated genome-scale metabolic network reconstructions for each of the clinical isolates. Metabolic network reconstructions combined with constraint-based analyses allow for a quantitative exploration of the functional repertoire and diversity of biological systems. The reconstruction of a genome-scale metabolic network involves the iterative process of synthesizing and validating existing biological data such as chemical reactions enabling metabolic functions, their associated enzymes, and gene-to-enzyme relationships. Genome annotation, biochemical data, and data from physiological experiments are all resources used to construct a network reconstruction. A stoichiometric matrix captures a detailed description of a biochemical network and provides a mathematical formalism to account for interactions of metabolic network components (43, 44). These methods have been used extensively to characterize the metabolism of antibiotic-resistant bacteria, bacterial adaptation during chronic infections, and the diversity of metabolic capabilities across many strains of microbial species, among other applications (10, 45, 46).

We used the previously published genome-scale metabolic network reconstruction of *P. aeruginosa* PA14, iPau21 (46), as the backbone on which the additionally annotated metabolic reactions were added to generate the draft reconstructions of the isolates. The *P. aeruginosa* PA14 metabolic model was also amended with additional annotated reactions that were absent in iPau21. Each of the models was checked for reaction mass balance.

### Contextualization of isolate metabolic models using transcriptomic data

Integrative Metabolic Analysis Tool (iMAT) (47) was used to contextualize GEMs by integrating gene expression data. The tool employs a flux balance analysis (FBA)-based framework to predict metabolic states that align with observed gene expression profiles. Genes were classified into high-expression, low-expression, and intermediate states based on their expression levels. Biomass production in *P. aeruginosa* iPau21 model was used as the objective function for gene essentiality analyses. Gene expression data were used to constrain fluxes through metabolic reactions, ensuring consistency with observed gene expression patterns.

### Analyses on the contextualized metabolic network models

The metabolic network reconstructions for each isolate were simulated in *in silico* synthetic cystic fibrosis medium (SCFM2) as described previously to generate 100 flux sample predictions for each model in the presence and absence of mucin. SCFM2 is a physiologically relevant medium that elicits more realistic metabolic behavior of the isolates in an infection setting. To compare the flux sampling results across isolates, a non-metric multidimensional scaling method was used. Here, each data point represents a functional metabolic snapshot of the flux sampling data. The data points are colored for each of the isolates, and differently shaped for SCFM2-mucin and SCFM2 + mucin growth medium. For estimating essential genes, a minimum biomass production threshold of 10% of wild-type biomass flux was assumed.

## Data Availability

Raw transcriptomic sequencing data generated in this study are available at https://www.ncbi.nlm.nih.gov/sra/PRJNA1305029. The Systems Biology Markup Language (sbml) version of all the reconstructions are available at https://anonymous.4open.science/r/PA_clinical_isolate_reconstructions/.

## SUPPLEMENTAL MATERIAL

Information S1 msystems.00580-26-s0001.xlsx Seven clinical *P. aeruginosa* isolates with relevant metadata.

Information S2 msystems.00580-26-s0002.xlsx Differentially expressed genes in the presence of mucin in each isolate that mapped to the reference PA14 strain.

Information S3 msystems.00580-26-s0003.xlsx Differentially expressed genes among the unmapped genes.

Information S4 msystems.00580-26-s0004.xlsx Reactions predicted to be essential for biomass production in the metabolic network models of the isolates.

Figure S1 msystems.00580-26-s0005.tiff Distribution of differentially overexpressed genes across the isolates.

Figure S2 msystems.00580-26-s0006.tiff Distribution of differentially underexpressed genes across the isolates.

Figure S3 msystems.00580-26-s0007.tiff Schematic of *de novo* transcriptomic pipeline.

Figure S4 msystems.00580-26-s0008.tiff Non-metric multidimensional scaling plot of the flux sampling simulations of the transcriptomic data-integrated models of the isolates.

Supplemental legends msystems.00580-26-s0009.docx Legends for supplemental material.

ASM does not own the copyrights to Supplemental Material that may be linked to, or accessed through, an article. The authors have granted ASM a non-exclusive, world-wide license to publish the Supplemental Material files. Please contact the corresponding author directly for reuse.
