# Supplementary figures and images for: Mucin-induced metabolic reprogramming in Pseudomonas aeruginosa clinical isolates
Source: mSystems. 2026 Jul 2;11(7):e00580-26. doi: 10.1128/msystems.00580-26 (PMC13387015; doi:10.1128/msystems.00580-26)

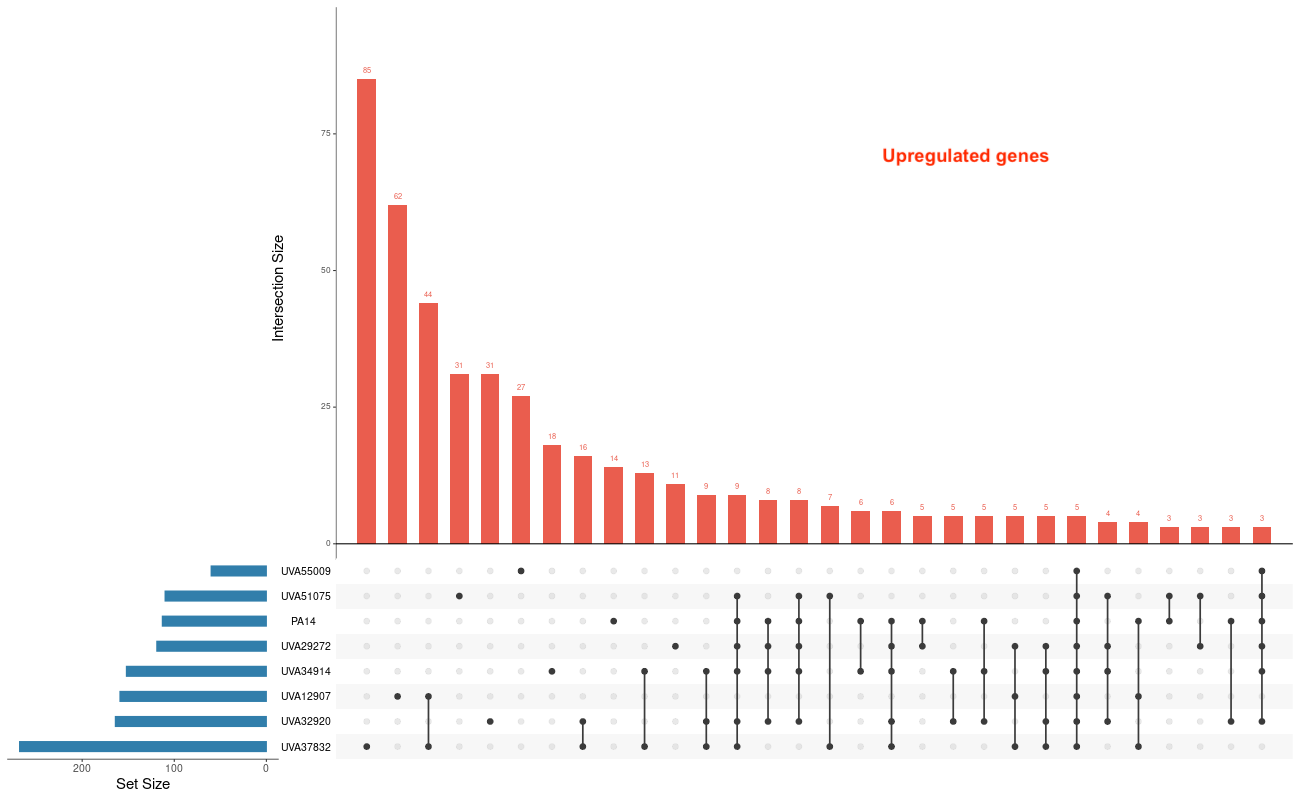

Supplement: Figure S1 — Distribution of differentially overexpressed genes across the isolates. [file msystems.00580-26-s0005.tiff]

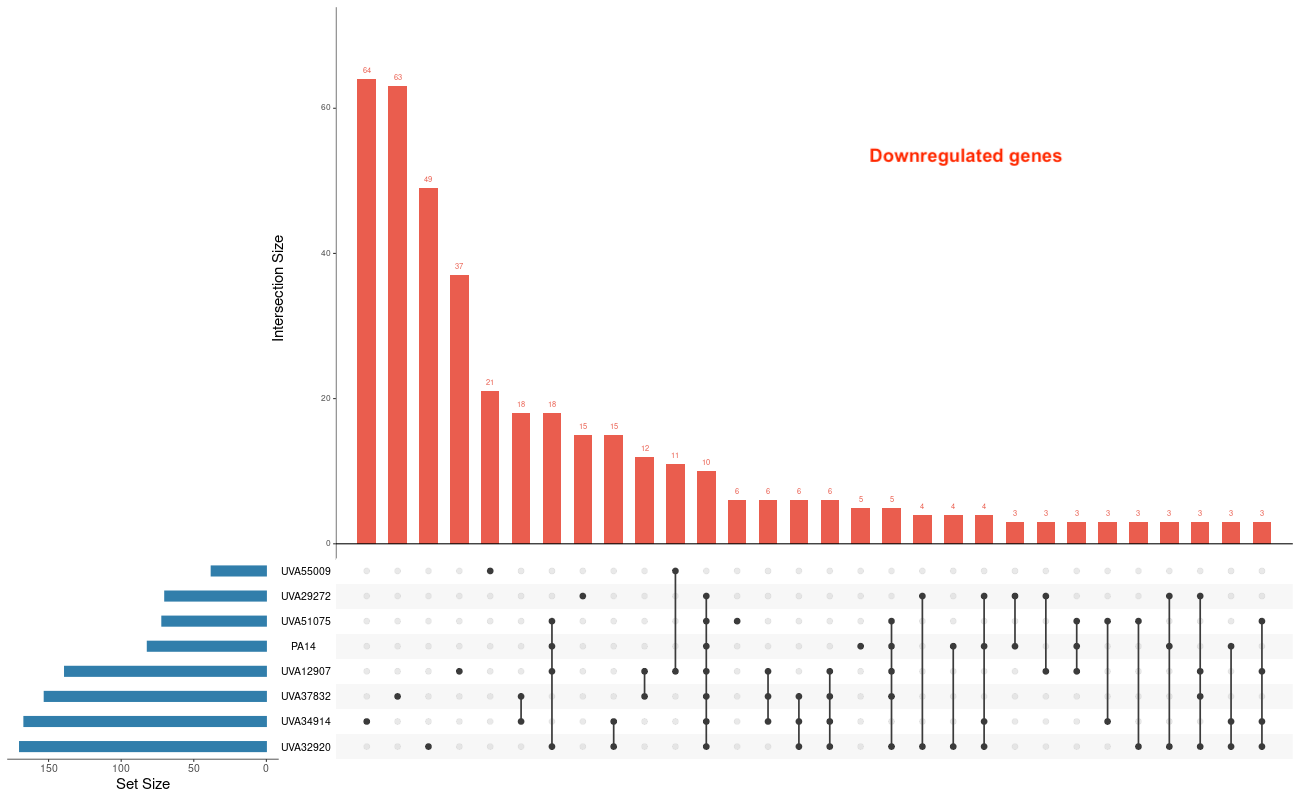

Supplement: Figure S2 — Distribution of differentially underexpressed genes across the isolates. [file msystems.00580-26-s0006.tiff]

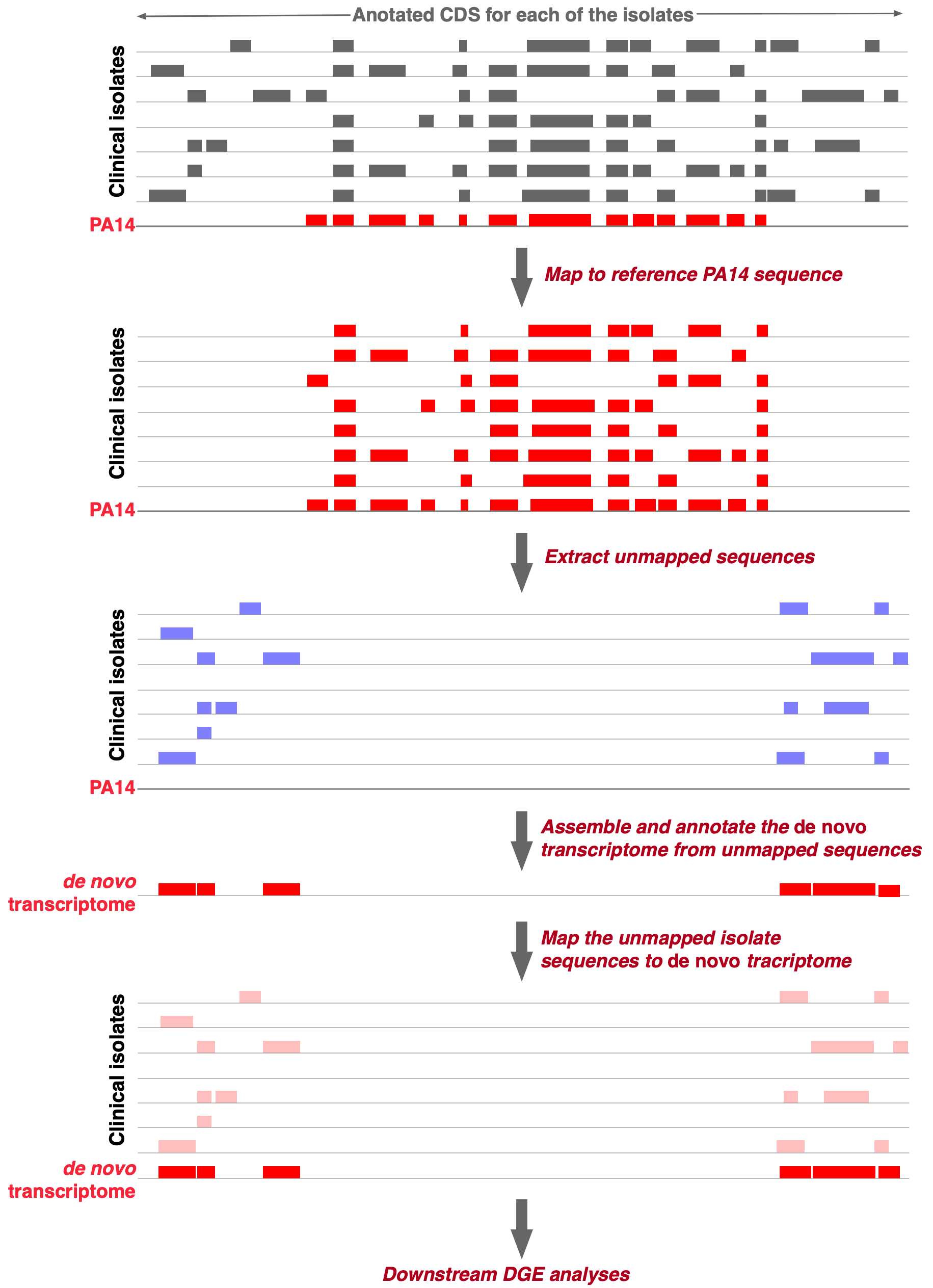

Supplement: Figure S3 — Schematic of de novo transcriptomic pipeline. [file msystems.00580-26-s0007.tiff]

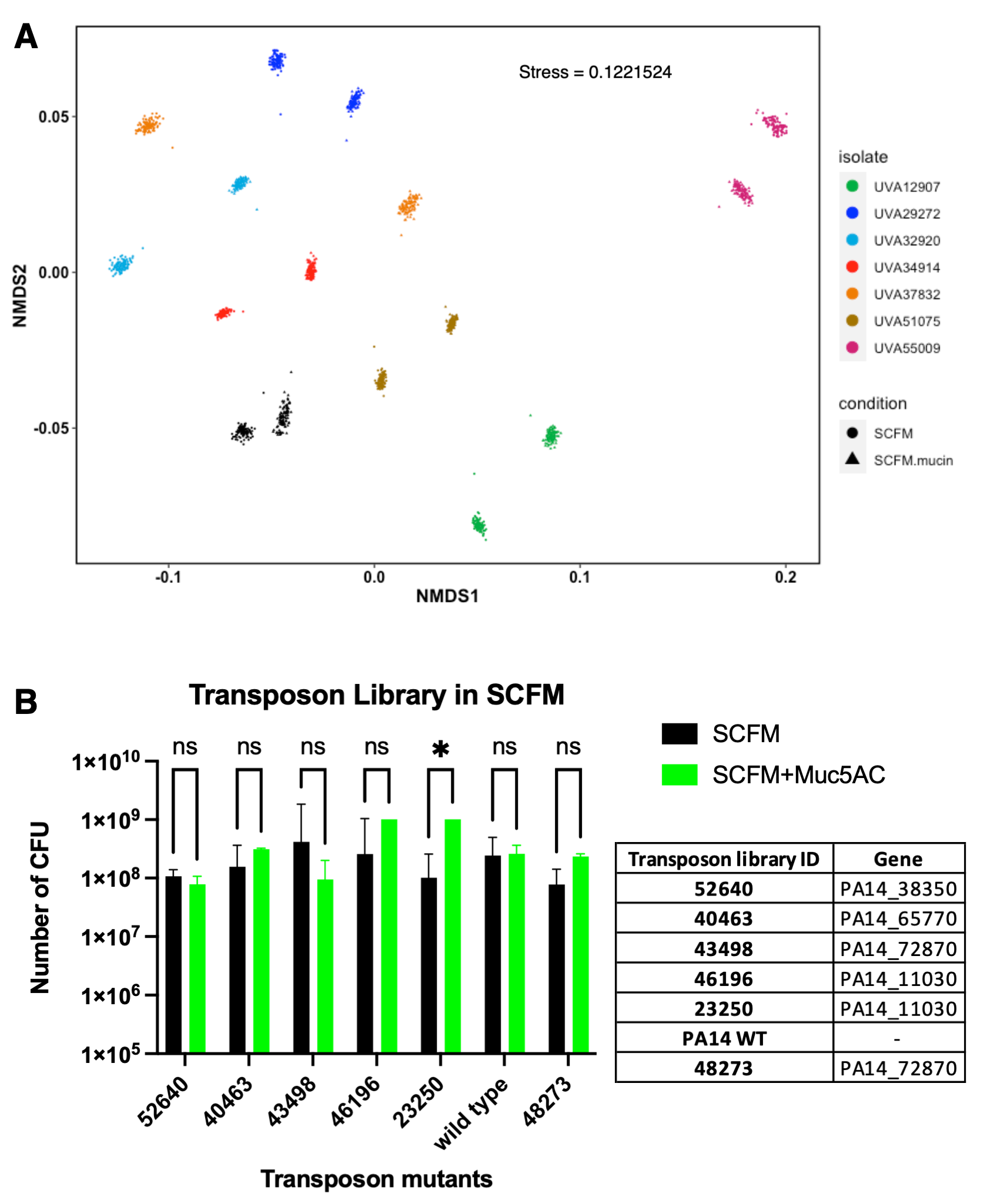

Supplement: Figure S4 — Non-metric multidimensional scaling plot of the flux sampling simulations of the transcriptomic data-integrated models of the isolates. [file msystems.00580-26-s0008.tiff]
