# Supplementary material for: Mucin-induced metabolic reprogramming in Pseudomonas aeruginosa clinical isolates
Source: mSystems. 2026 Jul 2;11(7):e00580-26. doi: 10.1128/msystems.00580-26 (PMC13387015; doi:10.1128/msystems.00580-26)
Supplement: Supplemental legends — Legends for supplemental material. [file msystems.00580-26-s0009.docx]

**Supplemental Material**

## Supplemental Information S1: List of seven clinical P. aeruginosa isolates with relevant metadata.

## Supplemental Information S2: List of the differentially expressed genes in the presence of mucin in each isolate that mapped to the reference PA14 strain.

Supplemental Information S3: List of the differentially expressed genes among the unmapped genes.

Supplemental Information S4: List of the reactions predicted to be essential for biomass production in the metabolic network models of the isolates.

Supplemental Figure 1: UpSet plot showing the distribution of differentially overexpressed genes across the isolates.

*Supplemental Figure 2:* UpSet plot showing the distribution of differentially underexpressed genes across the isolates.

*Supplemental Figure 3:* Schematic of *de novo* transcriptomic pipeline.

*Supplemental Figure 4:* Non-metric multidimensional scaling plot of the flux sampling simulations of the transcriptomic data-integrated models of the isolates.
